# Reversible Heart Failure After Anthracycline and Alkylating Agent Chemotherapy

**DOI:** 10.7759/cureus.99997

**Published:** 2025-12-24

**Authors:** Amresh Gul, Zahid Khan

**Affiliations:** 1 General Practice, GP Clinic, Brisbane, AUS; 2 Cardiology, University of South Wales, Pontypridd, GBR; 3 Cardiology, University of Buckingham, London, GBR; 4 Cardiology, Bart’s Heart Centre UK, London, GBR

**Keywords:** 2-dimensional echocardiography, anthracycline-induced cardiomyopathy, beta-blockers, guideline-directed medical therapy (gdmt), heart failure with reduced ejection fraction, mineralocorticoid receptor antagonist, pet ct scan, primary breast malignancy, sodium-glucose cotransporter-2 (sglt2) inhibitors, treatment of cardiomyopathy

## Abstract

Cardiotoxicity, including chemotherapy-induced heart failure (HF), remains a significant and well-recognised complication in modern oncologic practice. As cancer therapies evolve, treatment-related comorbidities continue to pose persistent challenges to patient outcomes and long-term survivorship. Among these, cytostatic-induced cardiotoxicity is particularly concerning due to its multifactorial and complex pathophysiology, involving oxidative stress, mitochondrial dysfunction, and direct myocardial injury. A comprehensive understanding of these mechanisms is essential for developing targeted preventive strategies and evidence-based therapeutic interventions.

We report a case of a 41-year-old Caucasian female who developed chemotherapy-induced cardiomyopathy, or heart failure with reduced ejection fraction (HFrEF), following treatment for right-sided breast cancer. Baseline echocardiogram showed normal biventricular function; however, her left ventricular ejection fraction (LVEF) declined to 39% following dual anthracycline-based chemotherapy, specifically doxorubicin and cyclophosphamide, for breast cancer. The patient was subsequently managed with guideline-directed medical therapy (GDMT). At six- and 12-month follow-up, her LVEF improved to 45% and 50-55%, respectively, demonstrating favourable cardiac recovery under optimised pharmacologic management.

## Introduction

Cardiotoxicity is a well-documented and potentially life-threatening complication of cancer chemotherapy, particularly with anthracycline-based regimens. As oncologic therapies have evolved, there has been growing awareness of their unintended cardiovascular effects, which can markedly affect both short- and long-term patient outcomes [1]. Among cytotoxic agents, anthracyclines - such as doxorubicin and epirubicin - remain cornerstone treatments for various solid and haematologic malignancies, including breast cancer. However, their clinical use is limited by dose-dependent myocardial injury, which can present as asymptomatic left ventricular dysfunction or progress to overt heart failure (HF) [2,3].

The pathophysiology of anthracycline-induced cardiotoxicity is multifactorial, involving oxidative stress, mitochondrial damage, and inhibition of topoisomerase IIβ in cardiomyocytes. These mechanisms culminate in apoptosis, fibrosis, and impaired contractility, often leading to a reduction in left ventricular ejection fraction (LVEF) [4,5]. Importantly, early identification and initiation of guideline-directed medical therapy (GDMT) have shown promise in reversing cardiac dysfunction and improving outcomes, underscoring the need for vigilant cardiac monitoring during and after chemotherapy [6].

HF is a complex clinical syndrome characterised by reduced cardiac output to meet the body's metabolic demands. It results in symptoms such as dyspnoea, fatigue, and fluid retention, and is a leading cause of hospitalisation in individuals over 65 years of age. HF can be broadly classified into HF with preserved ejection fraction (HFpEF) and HF with reduced ejection fraction (HFrEF), respectively, each with distinct pathophysiological mechanisms and therapeutic approaches. HF remains associated with significant morbidity and mortality despite significant advancement in both pharmacological and non-pharmacological therapy [7]. This report discusses the case of a patient with breast cancer who developed HFrEF following dual anthracycline therapy and subsequently demonstrated significant recovery under GDMT. The report underscores the importance of multidisciplinary care and prompt intervention in cardio-oncology.

## Case presentation

A 41-year-old female, previously healthy and a current smoker (four to five cigarettes per day), presented with a diagnosis of early-stage right breast cancer. She was diagnosed with invasive carcinoma of the breast of no special type, with associated ductal carcinoma in situ that was progesterone- and estrogen-receptor-positive but negative for human epidermal growth factor receptor 2 (HER2). 18F-fluorodeoxyglucose positron emission tomography/computed tomography (FDG PET/CT) did not reveal any evidence of distant metastasis. Her routine blood tests, including troponin and N-terminal pro-B-type natriuretic peptide (NT-proBNP), were normal, and she underwent a right-sided mastectomy followed by a lateral intercostal artery perforator (LICAP) flap reconstruction in June 2024 (Table 1).

**Table 1:** Serial blood test results HbA1c: glycated haemoglobin; LH: luteinising hormone; FSH: follicle-stimulating hormone, LDL: low density lipoprotein; HDL: high density lipoprotein; MCV: mean corpuscular volume; TSH: thyroid-stimulating hormone; TIBC: total iron-binding capacity; eGFR: estimated glomerular filtration rate; ALP: alkaline phosphatase; AST: aspartate aminotransferase; ALT: alanine aminotransferase; GGT: gamma-glutamyl transferase

Lab test	Levels: 17/10/2025	Levels: 06/03/2025	Levels: 28/08/2025	Reference range
Haemoglobin, g/L	151	156	154	135-175
MCV, fL	103	91	96	80-100
Platelets, 10^9^L	263	234	248	150-400
Haematocrit, L/L	0.48	0.54	0.48	0.40-0.54
TSH, mIU/L	2.0	2.5	2.2	0.3-3.5
Iron, umol/L	18	15	18	5-30
Transferrin, g/L	2.1	2.2	2.1	1.9-3.1
TIBC, umol/L	56	54	58	47-77
Ferritin, ug/L	111	78	72	30-300
Trans saturation, %	27	33	33	20-45
Sodium, mmol/L	139	138	139	135-145
Potassium, mmol/L	4.1	4.4	4.3	3.5-5.5
Chloride, mmol/L	106	109	108	95-110
Bicarbonate, mmol/L	24	25	19	20-32
Calcium, mmol/L	2.3	2.16	2.3	2.10-2.60
Phosphate, mmol/L	1.51	1.2	1.54	0.80-1.50
Urea, mmol/L	4.3	45.3	4.8	3.0-8.0
Creatinine, umol/L	65	61	65	60-110
eGFR, mL/min/1.73m²	90	90	90	>60
Random glucose, mmol/L	5.8	4.7	4.1	3.6-7.7
Total protein, g/L	67	768	64	66-83
Albumin, g/L	44	42	44	35-48
Total bilirubin, umol/L	6	9	9	<21
ALP, U/L	71	75	89	35-110
AST, U/L	17	18	23	10-40
ALT, U/L	17	16	17	5-40
GGT, U/L	21	22	15	5-40
Total cholesterol, mmol/L	5.8	5.8	6.4	<5.6
Triglycerides, mmol/L	2.0	1.8	3.7	<2.1
HDL, mmol/L	1.09	1.11	1.19	>0.89
LDL, mmol/L	3.9	3.9	3.7	<4.1
FSH, IU/L	-	23	45	
LH, IU/L	-	21	25	
Oestrodial, IU/L	-	249	<50	
Vitamin B12, pmol/L	510	381	391	>150
Folate, nmol/L	28	33	32	>7.0
Vitamin D, pmol/L	110	76	62	60-208
HbA1c, mmol/mol	5.4	5.4	5.5	<6.5

Baseline transthoracic echocardiography revealed a normal LVEF of 64% without any structural abnormalities. Adjuvant chemotherapy was initiated in July 2024, consisting of dose-dense doxorubicin and cyclophosphamide, followed by paclitaxel, and was completed in November 2024. The patient developed intermittent chest pain, dyspnea, and a non-productive cough during chemotherapy. Coronary CT angiography was unremarkable, with a calcium score of zero. A repeat PET-CT scan performed three months after mastectomy revealed only postsurgical changes, with no evidence of distant metastasis or disease recurrence.

Repeat echocardiography performed in December 2024 revealed a mildly dilated left ventricle with reduced systolic function and LVEF of 40-45%, with multiple regional wall motion abnormalities. Global longitudinal strain (GLS) was impaired at -16.9%, and multiple myocardial segments, including the mid anterolateral, apical lateral, basal interventricular septum, mid and apical inferior, basal and mid anterior, apical anterior, inferior-lateral, and mid anteroseptal segments, were hypokinetic (Videos 1-2). Holter monitoring results were unremarkable for arrhythmias. She was diagnosed with chemotherapy-induced cardiomyopathy and commenced on GDMT, including ramipril 2.5 mg, dapagliflozin 10 mg, spironolactone 25 mg, and bisoprolol 2.5 mg.

**Video 1:** Parasternal long axis echocardiographic view showing mild to moderate left ventricular systolic dysfunction

**Video 2:** Apical 4 chamber echocardiogram showing mild to moderate left ventricular systolic dysfunction

After six weeks of therapy, the patient reported marked improvement in symptoms. Her blood pressure was 121/85 mmHg, and her heart rate was 66 bpm and regular. Repeat echocardiography showed mild left ventricular dilation, improved LVEF of 50-55%, and resolution of regional wall motion abnormalities (Videos 3-5). Diastolic function was graded as 1, with normal filling pressures. She was advised to continue GDMT, and the doses of ramipril and bisoprolol were increased to 5 mg and 5 mg, respectively, while those of dapagliflozin and spironolactone were continued at the same doses. She completed 15 fractions of adjuvant radiotherapy in late January 2025, and her latest echocardiogram revealed a normal LVEF. She remains under cardiology and oncology follow-up and is stable, with no recurrence of the disease.

**Video 3:** Apical 4 chamber view of the echocardiography showing preserved left ventricular ejection fraction

**Video 4:** Parasternal long axis view showing preserved left ventricular ejection fraction

**Video 5:** Echocardiogram showing global longituidnal stress

## Discussion

Anthracycline-based chemotherapy, particularly doxorubicin, remains a cornerstone in the treatment of early-stage breast cancer, particularly in hormone receptor-positive, HER2-negative subtypes [2,3]. This case report illustrates the development of anthracycline-induced cardiomyopathy in a previously healthy 41-year-old female with clinical features consistent with HFrEF and subsequent recovery following timely initiation of GDMT. The common risk factors include cumulative anthracycline exposure, female sex, smoking, and the lack of cardioprotective agents such as dexrazoxane [5]. While coronary CT angiography excluded ischaemic disease, transthoracic echocardiography revealed a decline in LVEF from 64% to 39%, with regional wall motion abnormalities and GLS of -16.9%. These findings, along with a moderately dilated left ventricle and indexed left ventricular end-diastolic volume (LVEDV) of 71 ml/m², were consistent with non-ischaemic cardiomyopathy and early-stage HF. GLS is a sensitive marker of subclinical myocardial dysfunction and has demonstrated prognostic value in predicting chemotherapy-induced cardiotoxicity [8].

Management was initiated in accordance with international heart failure guidelines, incorporating angiotensin-converting-enzyme (ACE) inhibitor (ramipril), beta-blocker (bisoprolol), mineralocorticoid receptor antagonist (spironolactone), and SGLT2 inhibitor (dapagliflozin) [9]. This quadruple therapy has shown mortality and morbidity benefits in HFrEF, including in cancer survivors [7,9]. Within six weeks, the patient demonstrated symptomatic improvement and partial recovery of systolic function (EF 50%), with resolution of wall motion abnormalities and normalisation of filling pressures. This report underscores several key principles of cardio-oncology. Baseline and serial cardiac imaging, along with biomarkers such as troponin and NT-proBNP, are essential during anthracycline therapy for detecting early myocardial changes [10]. When initiated promptly, GDMT can reverse cardiotoxicity and improve outcomes in patients with HFrEF [10,11].

Multidisciplinary collaboration between oncology and cardiology is critical to optimise care and reduce long-term cardiovascular risk. Recent European Society of Cardiology (ESC) and European Society for Medical Oncology (ESMO) guidelines emphasise the importance of integrated cardio-oncology services, risk stratification, and biomarker-guided surveillance using troponin and NT-proBNP [12,13]. Imaging modalities such as strain echocardiography, cardiac MRI, and multimodality protocols are increasingly recommended for early detection and longitudinal monitoring of this condition [14]. As breast cancer survivorship continues to improve, the burden of cardiovascular complications is becoming increasingly relevant. Prophylactic strategies, including dexrazoxane in high-risk patients and adherence to cardio-oncology guidelines, may further mitigate cardiotoxic risk and enhance long-term outcomes.

## Conclusions

This case highlights the development of anthracycline-induced cardiomyopathy in a patient undergoing adjuvant chemotherapy for HER2-negative and hormone receptor-positive breast cancer. Despite initial cardiac stability, the patient developed symptomatic HFrEF following completion of dose-dense AC-T therapy. Prompt recognition, exclusion of ischaemic pathology, and initiation of GDMT led to significant improvement in both symptoms and cardiac function within six weeks. The report underscores the importance of baseline and serial cardiac surveillance, including echocardiography and GLS, in patients receiving potentially cardiotoxic regimens. It also reinforces the reversibility of chemotherapy-induced cardiomyopathy when managed early and appropriately. As cancer survivorship continues to rise, integrating cardio-oncology principles into routine oncologic care is essential to optimise long-term outcomes and preserve cardiovascular health.
